# Normalizing HDAC2 Levels in the Spinal Cord Alleviates Thermal and Mechanical Hyperalgesia After Peripheral Nerve Injury and Promotes GAD65 and KCC2 Expression

**DOI:** 10.3389/fnins.2019.00346

**Published:** 2019-04-10

**Authors:** Bihan Ouyang, Dan Chen, Xinran Hou, Tongxuan Wang, Jian Wang, Wangyuan Zou, Zongbin Song, Changsheng Huang, Qulian Guo, Yingqi Weng

**Affiliations:** ^1^Health Management Center, Xiangya Hospital of Central South University, Changsha, China; ^2^Department of Anesthesiology, Xiangya Hospital of Central South University, Changsha, China

**Keywords:** histone deacetylase 2, neuropathic pain, spinal cord, GAD65, KCC2

## Abstract

Neuropathic pain is a worldwide health concern with poor treatment outcomes. Accumulating evidence suggests that histone hypoacetylation is involved in development and maintenance of neuropathic pain. Thus, many natural and synthetic histone deacetylase (HDACs) inhibitors were tested and exhibited a remarkable analgesic effect against neuropathic pain in animals. However, studies evaluating specific subtypes of HDACs contributing to neuropathic pain are limited. In this study, using the chronic constriction injury (CCI) rat model, we found that mRNA and protein levels of HDAC2 were increased in the lumbar spinal cord of rats after sciatic nerve injury. Intrathecal injection of TSA, a pan-HDAC inhibitor, suppressed the increase in HDAC2 protein but not mRNA, and showed a dose-dependent pain-relieving effect. By introducing HDAC2-specific shRNA into the spinal cord via a lentivirus vector, we confirmed that HDAC2 mediates mechanical and thermal hyperalgesia after nerve injury. Further examination found two essential participants in neuropathic pain in the inhibitory circuit of the central nervous system: GAD65 and KCC2 were increased in the spinal cord of CCI rats after HDAC2 knockdown. Thus, our research confirmed that HDAC2 was involved in mechanical and thermal hyperalgesia induced by peripheral nerve injury. Furthermore, GAD65 and KCC2 were the possible downstream targets of HDAC2 in pain modulation pathways.

## Introduction

Neuropathic pain is pain caused by a lesion or disease in the somatosensory system (Jensen et al., 2011). Symptoms include continuous and episodic spontaneous pain, hypersensitivity (overreaction to painful stimuli), and/or allodynia (pain from normally non-painful stimuli) evoked by mechanical, thermal, and/or cold stimuli. Existing treatments for neuropathic pain have been largely ineffective, providing inadequate pain relief and resulting in multiple adverse effects (Alles and Smith, 2018). Thus, a large number of studies have aimed to elucidate the underlying mechanisms of neuropathic pain and to develop new therapeutic strategies.

Altered expression of a variety of genes contributes to immediate and long-term molecular and structural changes in neurons and glia after peripheral nerve injury, which can lead to onset and maintenance of neuropathic pain (Alles and Smith, 2018). Multiple epigenetic mechanisms, such as DNA methylation/demethylation, histone modifications, and non-coding RNAs, can manipulate gene expression without changing the primary DNA sequence. Histone deacetylases (HDACs) are a large group of enzymes that catalyze disassociation of acetyl groups from the N-tail of histones in chromatin. HDACs function as transcriptional repressors by compacting the structure of chromatin and hindering access of transcriptional factors to gene promoters. In humans, there are 18 HDACs classified into four groups based on their structures and catalytic activity. Class I (including HDACs 1, 2, 3, and 8), II (including HDACs 4, 5, 7, 5, 9, and 10), and IV (HDAC 11) require zinc as a cofactor. Class III HDACs (also known as sirtuins) require nicotinamide adenine dinucleotide as a cofactor. Disruption of HDAC expression or function contributes to many pathological conditions, such as carcinoma, neurodegenerative disease, psychological disease, and inflammation (Falkenberg and Johnstone, 2014; Abend and Kehat, 2015). Recently, the analgesic effect of many HDAC inhibitors was identified in clinical and animal studies, implying involvement of HDACs in pathological pain (Niesvizky et al., 2011; Vojinovic and Damjanov, 2011; Zhang et al., 2011; Denk et al., 2013). Due to lack of specific inhibitors, the role of different HDAC subtypes in pain processing is not well characterized. In our previous studies, we identified the modulating effect of HDAC2 in bone cancer pain by utilizing gene-interfering techniques. Whether HDAC2 is involved in other kinds of pathological pain has not been characterized (Hou et al., 2018). To examine changes in HDAC2 expression after peripheral nerve injury and to explore its role in neuropathic pain, we conducted the present study.

## Materials and Methods

### Animals

We used adult male Sprague–Dawley rats from the animal experiment center of Xiangya Medicine School (Central South University, Changsha, Hunan, China), where all animal experiments were conducted. Rats were housed under standard conditions (temperature at 22 ± 2°C, light/dark cycle of 12/12 h) before surgery for at least 1 week. At the time of surgery, animals weighed between 220 and 250 g. All procedures involving use of animals were in accordance with the guideline of the International Association for the Study of Pain (IASP) and were approved by Xiangya Medical College of Central South University Animal Care and Use Committee (Changsha, China) (the approved code was 201503373).

### Chronic Constriction Injury (CCI) Model

Animals were anesthetized by isoflurane inhalation, and chronic constriction injury (CCI) was induced following the surgical procedure as previously described (Zhu et al., 2014). In brief, the left sciatic nerve was exposed above the branching point at mid-thigh level and loosely ligated with 4-0 chromic gut for four times at intervals of 1 mm between each ligation. In the sham group, the sciatic nerve was exposed without ligation. All surgical procedures were performed by the same individual to avoid variability.

### Behavior Measurements

Before each test, the rats were placed in a Plexiglas box on the mesh grid for a 30 min acclimation period. Mechanical thresholds in rats were tested with von Frey probes (Stoelting, United States) as previously reported (Xu et al., 2018). Paw withdrawal mechanical threshold (PWMT) was recorded as the force of von Frey filament which induced at least three positive responses out of five applications. Thermal sensitivity thresholds were tested by Hargreaves Tes7370 (Ugo Basile, Italy) (Ding et al., 2017). Paw withdrawal thermal latency (PWTL) was recorded as the duration of radiant heat stimulation which was turned off automatically by paw withdrawal (cutoff time was 30 s to avoid tissue damage). PWMT and PTWL were measured in triplicate and averaged with an interval of 5 min. The motor function of rats receiving TSA or lentivirus injection was tested at day 10 or 14 after CCI, respectively, using rotarod test following the protocol we have described previously (Dai et al., 2019).

### Drugs and Intrathecal Injection

TSA, a pan-HDAC inhibitor was dissolved in 5% dimethyl sulfoxide (DMSO) in saline solution and stored at −20°C. TSA was injected for 3 consecutive days with the first injection on the seventh day after CCI surgery to ensure establishment of neuropathic pain. A 10 μL intrathecal injection was administered as reported previously under isoflurane anesthesia (Mo et al., 2018). Sham-operated rats and DMSO-treated rats received 5% DMSO, and TSA-treated rats received TSA doses of either 2 or 10 μg (one dose per day).

### Lentivirus

HDAC2-specific RNAi was designed and synthesized by Shanghai Genechem Co., Ltd. (Shanghai, China). The sequence of HDAC2-specific RNAi was 5′-CCGTGAAGCTGAACCGTCA-3′. U6-MCS-Ubi-EGFP was used as the frame structure and GV118 as the vector. The package of lentivirus carrying the RNA interfering sequence (LV-shRNA-HDAC2) or control sequence (LV-NC) was transfected in 293 T cells. The final titer was approximately 1 × 10^9^ TU (transducing units)/mL. Recombinant lentivirus (10 μL) was intrathecally injected on the seventh day after CCI surgery through an intrathecal catheter. The infection efficiency was evaluated via GFP (Green Fluorescent Protein) signal detected using a Leica DM5000 fluorescence microscopy.

### Sample Preparation

At the predetermined time points, rats were sacrificed after behavioral tests and the L4-6 lumbar spinal cord tissues were collected. Samples for RT-PCR and western blot experiments were snap-frozen in liquid nitrogen and then stored at −80°C. Samples used for immunofluorescence imaging were post-fixed with 4% paraformaldehyde for 6 h and dehydrated with 15–30% sucrose overnight at 4°C and kept at −20°C.

### Immunofluorescence

The lumbar spinal cord was transected into 10-μm-thick sections using a cryostat and 8–10 slices were mounted directly onto a glass slide (Fisher Scientific). Sections were washed on the glass slides in 0.01 M paraformaldehyde/PBS and then blocked with 3% donkey serum for 1 h. Sections were incubated overnight with a mouse antibody against HDAC2 (1:100, Abcam), and rabbit antibodies against Iba-1 (1:500, Abcam), NeuN (1:300, Abcam), or GFAP (1:600, Abcam). The sections were then washed three times with PBS, then incubated in the dark for 2 h with a donkey anti-mouse red fluorescent-antibody (1:200, Jackson) or donkey anti-rabbit green fluorescent-antibody (1:200, Jackson). PBS was used instead of the primary antibody as a negative control. Immunofluorescence was visualized and digitally captured using a Leica DM5000 fluorescence microscope.

### Western Blot

Frozen tissues were homogenized and proteins were extracted using a nucleoprotein and cytoplasmic protein extraction kit (Keygen Biotech, China); 30 μg of protein was mixed with SDS sample buffer. Proteins were separated on standard sodium dodecyl sulfate-polyacrylamide gel electrophoresis (8–10% gels) then transferred onto 0.45-μm polyvinylidene fluoride membranes (Millipore, United States). Membranes were blocked in 5% milk for 1 h and incubated overnight at 4°C with the following primary antibodies: mouse anti-HDAC2 (1:600, Abcam, United States), rabbit anti-GAPDH (1:500, Goodhere, China), and mouse anti-β-tubulin (1:1000, Beyotime, China). Membranes were incubated with peroxidase-conjugated secondary antibodies (1:5000, Jackson, United States) for 1.5 h at a room temperature about 25°C. Proteins were detected using a ChemiDoc luminescence system (Bio-Rad).

### Real-Time Fluorescent Quantitative PCR (RT-PCR)

Total RNA was extracted and purified from lumbar spinal cord tissues using Trizol reagent (Invitrogen, United States). Reverse transcription was performed using an All-in-One cDNA Synthesis Kit (GeneCopoeia, United States). Twenty microliters of standard qPCR reactions were run on a ViiA 7 qPCR system. The following primers were used (5′-3′):

HDAC2: forward_TGGGCTGCTTCAACCTAA CT, reverse_TCCAACATCGAGCAACATTC;
KCC2: forward_AAGGACCCCCGCATACAAAG, reverse_GACAGAGCCCACAATGGTCA
Gad65: forward_CCTTTCCTGGTGAGTGCCACAGC, reverse_TTTGAGAGGCGGCTCTTCTCTC

### Statistical Analysis

All data were presented as mean ± SEM. Data were tested for the assumptions of normal distribution and equal variances before further statistical analysis. PWMT and PWTL were analyzed using repeated measures ANOVA, and multiple comparisons between groups at each time point were conducted using Bonferroni’s post-test. For western blot and PCR data, one-way ANOVA with Bonferroni’s post-test or Kruskal–Wallis test with Dunn’s post-test were used (detailed statistical information for all data is summarized in section “Results”).

## Results

### Development of Mechanical and Thermal Hypersensitivity After Sciatic Nerve Injury

**FIGURE 1:**
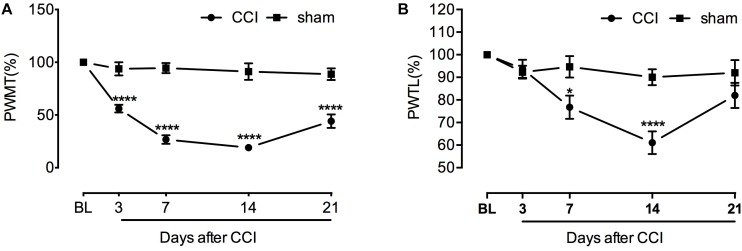
Chronic constriction of the sciatic nerve caused mechanical and thermal hyperlagesia in rats. **(A)** Decreased mechanical threshold was observed at the third day after CCI and lasted to the end of behavioral testing with slight recovery (*n* = 6 per group). ^∗∗∗∗^*P* < 0.0001, significantly different compared with the sham group; CCI, chronic constriction injury; BL, baseline; PWMT, paw withdrawal mechanical threshold. **(B)** Increased sensitivity to heat stimulus started from the seventh day after sciatic nerve injury and remained until day 14. Thermal threshold recovered at the end of behavioral testing. ^∗^*p* < 0.05, ^∗∗∗∗^*p* < 0.0001, significantly different compared with the sham group; PWTL, paw withdrawal thermal latency.

After sciatic nerve ligation, CCI rats showed pain sensitizing behaviors such as paw protection, paw licking, and dorsiflexion (data not shown). Starting from the third day after operation, CCI rats developed significant mechanical hyperalgesia which lasted to the end of behavioral testing (Figure 1A). Developing later and recovering faster than PWMT, paw withdrawal latency to noxious heat stimuli was significantly decreased between day 7 and 14 (Figure 1B). [*For mechanical threshold*: between different groups: *F*_(1,10)_ = 97.66, *P* < 0.0001; between different time points: *F*_(4,40)_ = 37.87, *P* < 0.0001; interaction: *F*_(4,40)_ = 25.51, *P* < 0.0001; multiple comparisons: sham group vs. CCI group, on day 3, 93.857 ± 6.279 vs. 56.080 ± 3.674, *t*_(50)_ = 5.550, *p* < 0.0001; day 7, 94.460 ± 4.872 vs. 26.716 ± 4.078, *t*_(50)_ = 9.952, *p* < 0.0001; day 14, 91.214 ± 7.934 vs. 19.067 ± 2.020, *t*_(50)_ = 10.60, *p* < 0.0001; day 21, 88.773 ± 5.575 vs. 44.199 ± 6.348, *t*_(50)_ = 6.548, *p* < 0.0001. *n* = 6 for each group. *For thermal threshold*: between different groups: *F*_(1,10)_ = 6.126, *P* = 0.0328; between different time points: *F*_(4,40)_ = 17.90, *P* < 0.0001; interaction: *F*_(4,40)_ = 8.772, *P* < 0.0001; multiple comparisons: sham group vs. CCI group, day 7, 94.649 ± 4.727 vs. 76.775 ± 5.117, *t*_(50)_ = 3.034, *p* = 0.0191; day 14, 90.013 ± 3.580 vs. 61.078 ± 5.037, *t*_(50)_ = 4.911, *p* < 0.0001, *n* = 6 for each group.]

### HDAC2 Expression Was Increased Time-Dependently in the Spinal Cord of CCI Rats

**FIGURE 2:**
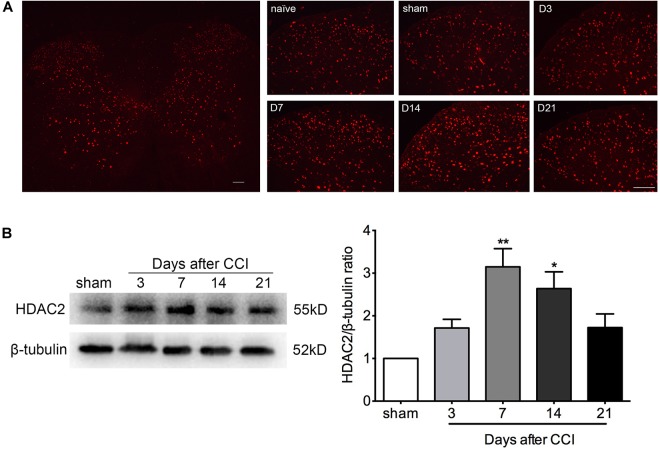
HDAC2 expression was enhanced in a time-dependent manner in the lumbar spinal cord of CCI rats. **(A)** Immunofluorescent signal of HDAC2 (red) detected in the whole lumbar spinal cord section and the ipsilateral dorsal horn of rats. D3: 3 days after CCI; D7: 7 days after CCI; D14: 14 days after CCI; D21: 21 days after CCI; scale bar = 100 μm. **(B)** Western blot analysis showed that HDAC2 protein levels were altered in a time-dependent manner. A significant increase was detected on days 7 and 14 after CCI; *n* = 4 per group, ^∗^*p* < 0.05, ^∗∗^*p* < 0.01, compared to the Sham group.

Immunofluorescent staining demonstrated a scattered distribution of HDAC2 in the gray matter of lumbar spinal tissue, with a relatively higher density in the dorsal horn. The abundance of HDAC2 positive cells in the ipsilateral dorsal horn was elevated in a time-dependent manner after CCI (Figure 2A and Supplementary Figures S2, S3). Quantification of HDAC2 protein by western blot analysis confirmed a time-dependent upregulation of HDAC2 protein in the lumbar spinal cord, which was parallel to the time course of decrements in paw withdrawal threshold. A significant change in HDAC2 protein was detected at days 7 and 14 after CCI surgery (Kruskal–Wallis statistic = 14.24, *P* = 0.0066; multiple comparisons: sham group vs. CCI group, on day 7, 1.0 vs. 3.150 ± 0.4265, *p* = 0.0020; day 14, 1.0 vs. 2.639 ± 0.3925, *p* = 0.0193; *n* = 4 for each group) (Figure 2B).

### HDAC2 Was Mainly Expressed in Spinal Cord Neurons

**FIGURE 3:**
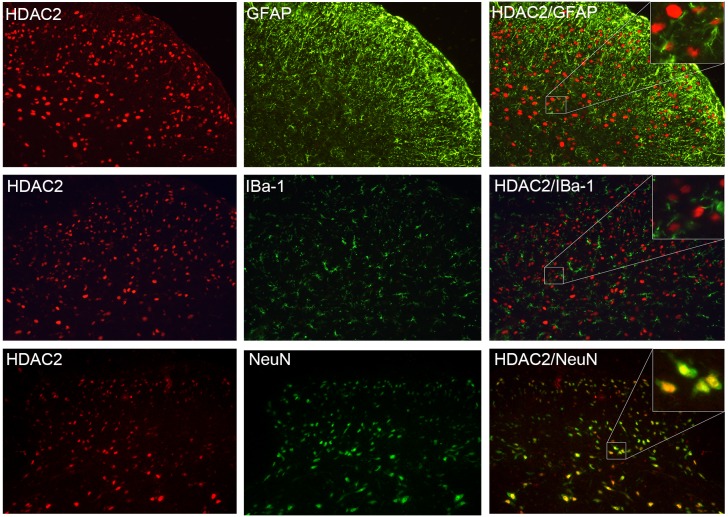
HDAC2 was primarily expressed in neurons. HDAC2 signal (red) was highly co-localized with NeuN (marker of the neuron, green). There was no overlap between HDAC2 and the astroglial marker GFAP (green), or HDAC2 with the microglial marker IBa-1 (green).

Double immunofluorescent staining images revealed that the majority of the HDAC2 signals overlapped with NeuN (a marker of neurons), but not with GFAP (an astroglial marker) or Iba-1 (a microglial marker) (Figure 3), however, as HDAC2 was predominantly expressed in the nucleus (Hou et al., 2018) while GFAP and Iba-1 were expressed in the cytoplasm, it is hard to detect an overlap between them. Because a few HDAC2 signals were also detected in some NeuN negative cells, the distribution of HDAC2 in astroglia and microglia could not be excluded.

### Intrathecal TSA Attenuated Mechanical and Thermal Hyperalgesia in CCI Rats in a Dose-Dependent Manner

**FIGURE 4:**
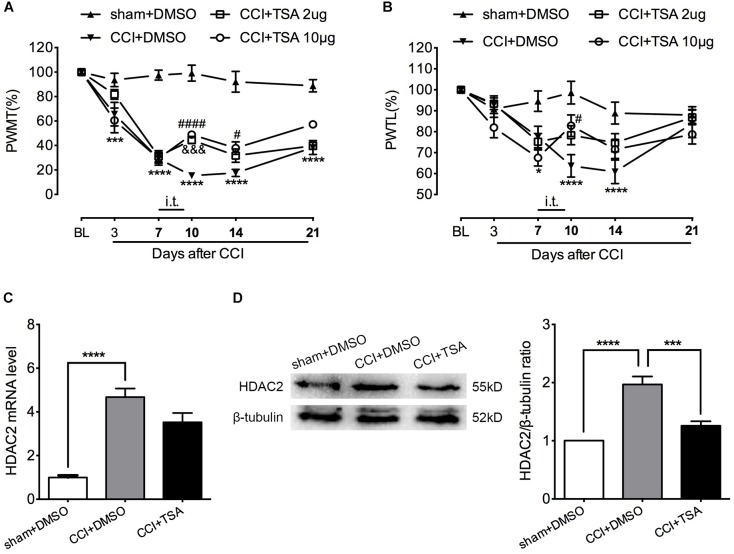
Dose-dependent effect of TSA on mechanical and thermal threshold of CCI rats. **(A)** Intrathecal injection of TSA (once per day for three consecutive days starting at the seventh day) partially reversed the decrease in mechanical threshold in CCI rats. The analgesic effect was obvious on days 10 and 14 post-injury in the 10 μg group (*n* = 6 per group). In the 2 μg group, the effect was only significant on day 10 post-injury. ^∗∗∗^*p* < 0.001, ^∗∗∗∗^*P* < 0.0001, CCI+DMSO vs. sham+DMSO; ^#^*p* < 0.05, ^####^*p* < 0.0001, CCI+TSA 10 μg vs. CCI+DMSO; ^&&&^*p* < 0.001, CCI+TSA 2 μg vs. CCI+DMSO; BL, baseline; PWMT, paw withdrawal mechanical threshold. **(B)** Repeated daily intrathecal injection of TSA partially reversed the decreased thermal threshold at day 10 after CCI in the 10 μg group (*n* = 6 per group). ^∗^*p* < 0.05, ^∗∗∗∗^*p* < 0.0001, CCI+DMSO vs. sham+DMSO; ^#^*p* < 0.05, CCI+TSA 10 μg vs. CCI+DMSO; PWTL, paw withdrawal thermal latency. **(C,D)** Repeated intrathecal injection of TSA (10 μg) daily suppressed HDAC2 protein expression but not mRNA level in the lumbar spinal cord of CCI rats; *n* = 4 per group, ^∗∗∗^*p* < 0.001, ^∗∗∗∗^*p* < 0.0001.

To evaluate potential HDAC2 involvement in neuropathic pain, we used the pan-HDAC inhibitor TSA to block spinal HDACs function by daily intrathecal injection at days 7, 8, and 9 after CCI surgery, when hyperalgesia was apparent and stable. The TSA injection did not affect the motor function of rats according to the rotarod test (Supplementary Figure S1). As shown in Figure 4A, among CCI rats receiving 2 μg TSA, the mechanical threshold was significantly higher at day 10. However, the analgesic effect was temporary and dissipated by days 14 and 21. Among CCI rats that received the higher dose of TSA (10 μg in 10 μL), mechanical hyperalgesia was partially reversed at day 10 and the effect persisted until day 14. For thermal hyperalgesia, the analgesic effect was only observed at day 10 in rats that received 10 μg of TSA (Figure 4B). [*For mechanical threshold*: between different groups: *F*_(3,20)_ = 53.96, *P* < 0.0001; between different time points: *F*_(5,100)_ = 97.59, *P* < 0.0001; interaction: *F*_(15,100)_ = 12.73, *P* < 0.0001; multiple comparisons: CCI + DMSO group vs. CCI + TSA 10 μg group, *n* = 6 for each group, on day 10, 15.400 ± 2.439 vs. 48.958 ± 2.542, *t*_(120)_ = 4.757, *p* < 0.0001; day 14, 17.546 ± 2.937 vs. 37.857 ± 5.116, *t*_(120)_ = 2.879, *p* = 0.0284. CCI + DMSO group vs. CCI + TSA 2 μg group, *n* = 6 for each group, on day 10, 15.400 ± 2.439 vs. 44.543 ± 2.968, *t*_(120)_ = 4.131, *p* = 0.0004. *For thermal threshold*: between different groups: *F*_(3,20)_ = 4.596, *P* = 0.0133; between different time points: *F*_(5,100)_ = 28.65, *P* < 0.0001; interaction: *F*_(15,100)_ = 4.663, *P* < 0.0001; multiple comparisons: CCI + DMSO group vs. CCI + TSA 10 μg group, *n* = 6 for each group, on day 10, 63.742 ± 5.355 vs. 82.855 ± 5.142, *t*_(120)_ = 3.139, *p* = 0.0128.]

### TSA Decreased HDAC2 Protein Level but Not mRNA Abundance

To evaluate the effect of TSA on HDAC2 expression, we administrated 10 μg of TSA (or 10% DMSO as vehicle) intrathecally to CCI rats for 3 consecutive days from days 7 to 9 after CCI. The lumbar spinal cord was harvested at day 10 and HDAC2 mRNA and protein levels were measured. As shown in Figure 4C,D, HDAC2 mRNA levels were robustly elevated in both CCI + DMSO and CCI + TSA groups, and TSA treatment slightly blunted CCI-induced up-regulation of HDAC2 (not statistically significant). Similar to the results obtained in CCI rats in previous experiments, the abundance of HDAC2 protein in CCI + DMSO rats was dramatically increased at day 10, and this upregulation was reversed by 10 μg TSA treatment. [*For HDAC2 mRNA*: *F*_(2,9)_ = 30.68, *P* < 0.0001; multiple comparisons: 1 ± 0.09732 in sham + DMSO group vs. 4.674 ± 0.3952 in CCI + DMSO group, *t*_(9)_ = 7.656, *p* < 0.0001; 4.674 ± 0.3952 in CCI + DMSO group vs. 3.527 ± 0.4239 in CCI + TSA group, *t*_(9)_ = 2.391, *p* = 0.0810; *n* = 4 for each group. *For HDAC2 protein*: *F*_(2,9)_ = 29.16, *P* = 0.0001; multiple comparisons: 1 in sham + DMSO group vs. 1.968 ± 0.1399 in CCI + DMSO group, *t*_(9)_ = 7.374, *p* = 0.0001; 1.968 ± 0.1399 in CCI + DMSO group vs. 1.258 ± 0.07908 in CCI + TSA group, *t*_(9)_ = 5.406, *p* = 0.0009; *n* = 4 for each group.]

### HDAC2 Knockdown Attenuated Mechanical and Thermal Hyperalgesia in CCI Rats

**FIGURE 5:**
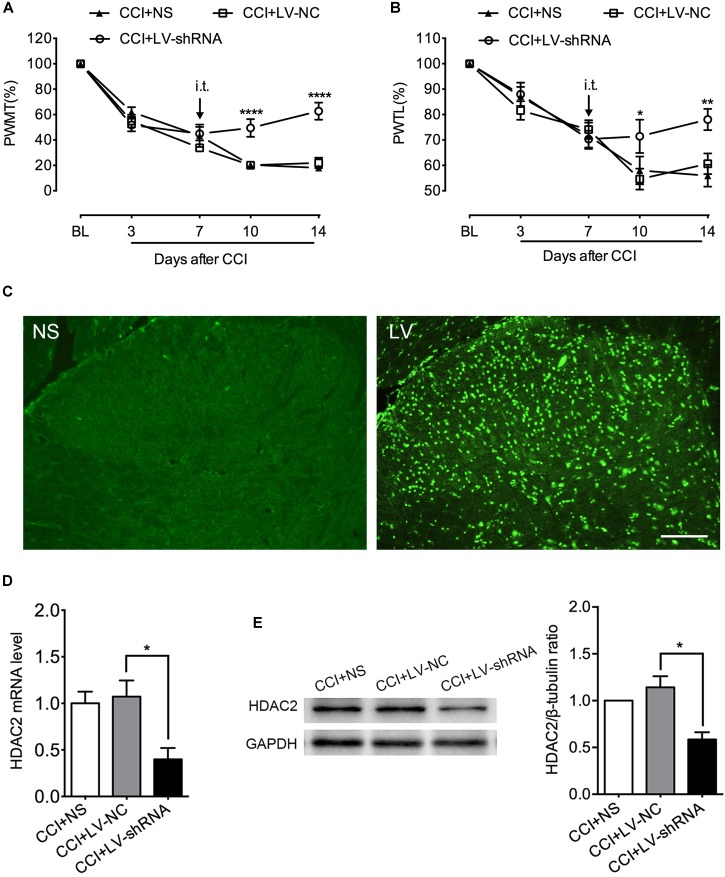
Intrathecal administration of LV-shRNA-HDAC2 repressed HDAC2 mRNA and protein expression and alleviated mechanical and thermal hyperalgesia in CCI rats. **(A,B)** Intrathecal administration of LV-shRNA-HDAC2 partially reversed the mechanical and thermal hyperalgesia in CCI rats since day 10 post-injury, and the analgesic effect lasted to the end of behavioral tests (*n* = 6 per group). ^∗^*p* < 0.05, ^∗∗^*P* < 0.01, ^∗∗∗∗^*P* < 0.0001, CCI+LV-shRNA vs. CCI+LV-NC; BL, baseline; PWMT, paw withdrawal mechanical threshold; PWTL, paw withdrawal thermal latency. **(C)** Successful transfection with lentivirus was demonstrated by GFP signal (green) detected by immunofluorescent imaging of the lumbar spinal cord. NS: rats receiving normal saline; LV: rats with lentivirus injection, scale bar = 100 μm. **(D,E)** Intrathecal injection of LV-shRNA-HDAC2 decreased HDAC2 mRNA and protein abundance in the lumbar spinal cord of CCI rats; *n* = 4 per group, ^∗^*p* < 0.05.

To further determine the specific role of HDAC2 in neuropathic pain, we intrathecally injected lentivirus carrying HDAC2-shRNA encoding genes to knock down spinal HDAC2. GFP immunofluorescent signal suggested a successful lentivirus transfection (Figure 5C). PCR and western blot analysis demonstrated successful knockdown of HDAC2 in the lumbar spinal cord (Figure 5D,E). HDAC2 knockdown did not alter the motor function of rats (Supplementary Figure S1). As shown in Figure 5A,B, HDAC2-shRNA alleviated mechanical and thermal hyperalgesia induced by CCI surgery, and rats that were infected with lentivirus carrying scrambled genes showed similar pain behavior as their cohorts that received intrathecal saline. [*For HDAC2 mRNA*: *F*_(2,9)_ = 6.758, *P* = 0.0161; multiple comparisons: 1.072 ± 0.1734 in CCI + LV-NC group vs. 0.3999 ± 0.1206 in CCI + HDAC-shRNA group, *t*_(9)_ = 3.348, *p* = 0.0257; *n* = 4 for each group. *For HDAC2 protein*: Kruskal–Wallis statistic = 8.290, *P* = 0.0026; multiple comparisons: 1.142 ± 0.1186 in CCI + LV-NC group vs. 0.5854 ± 0.07738 in CCI + LV-shRNA group, *p* = 0.0156; *n* = 4 for each group. *For mechanical threshold*: between different groups: *F*_(2,15)_ = 9.025, *P* = 0.0027; between different time points: *F*_(4,60)_ = 154.8, *P* < 0.0001; interaction: *F*_(8,60)_ = 9.695, *P* < 0.0001; multiple comparisons: CCI + LV-NC group vs. CCI + HDAC2-shRNA group, *n* = 6 for each group, on day 10, 20.123 ± 2.646 vs. 49.444 ± 6.980, *t*_(75)_ = 4.597, *p* < 0.0001; day 14, 21.966 ± 4.062 vs. 62.778 ± 6.786, *t*_(75)_ = 6.399, *p* < 0.0001. *For thermal threshold*: between different groups: *F*_(2,15)_ = 7.851, *P* = 0.0046; between different time points: *F*_(4,60)_ = 43.56, *P* < 0.0001; interaction: *F*_(8,60)_ = 2.059, *P* = 0.0543; CCI + LV-NC group vs. CCI + HDAC2-shRNA group, *n* = 6 for each group, on day 10, 54.623 ± 4.117 vs. 71.444 ± 6.562, *t*_(75)_ = 2.953, *p* = 0.0126; day 14, 60.626 ± 4.100 vs. 78.057 ± 4.207, *t*_(75)_ = 3.060, *p* = 0.0092.]

### HDAC2 Knockdown Increased Expression of GAD65 and KCC2 mRNA

**FIGURE 6:**
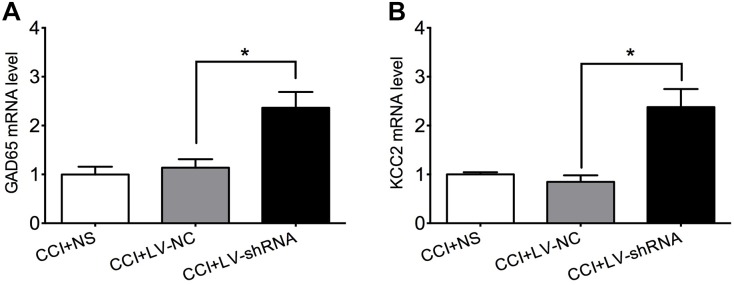
Knockdown of HDAC2 upregulated GAD65 **(A)** and KCC2 **(B)** mRNA in the lumbar spinal cord of CCI rats. ^∗^*p* < 0.05, CCI+LV-shRNA vs. CCI+LV-NC.

GAD65 and KCC2 are promising targets under the epigenetic modulation of HDAC2 (Zhang et al., 2011; Shen et al., 2014; Hou et al., 2018). They are indispensable components of the spinal inhibitory circuitry which are impaired under neuropathic pain. Rebooting their expression relieves mechanical and thermal hyperalgesia induced by peripheral nerve injury effectively (Moore et al., 2002; Coull et al., 2003; Kim et al., 2009; Lorenzo et al., 2014). As hypothesized, GAD65 and KCC2 mRNA levels were similar in the lumbar spinal cord of CCI rats that received normal saline and negative control lentivirus, but significantly elevated after treatment with LV-shRNA-HDAC2, which confirmed the suppressive modulation of HDAC2 to these two molecules (Figure 6). [*For GAD65*: *F*_(2,9)_ = 10.71, *P* = 0.0042; multiple comparisons: 1.138 ± 0.1724 in CCI + LV-NC group vs. 2.366 ± 0.3212 in CCI + HDAC-shRNA group, *t*_(9)_ = 3.778, *p* = 0.0131; *n* = 4 for each group. *For KCC2*: Kruskal–Wallis statistic = 7.731, *P* = 0.0066; multiple comparisons: 0.8482 ± 0.1337 in CCI + LV-NC group vs. 2.377 ± 0.3727 in CCI + LV-shRNA group, *p* = 0.0244; *n* = 4 for each group.]

## Discussion

Under pressure of endogenous or exogenous environmental changes, epigenetic modulation can act as an adaptive mechanism. However, these modifications can also exert a detrimental effect and lead to pathological conditions (Zhang and Ren, 2016; Niederberger et al., 2017). After nerve injury, the balance between histone acetylation/deacetylation in the peripheral and central nervous system can be disturbed. Though controversies still exist, upregulated HDACs and resultant downregulation of histone acetylation were believed to facilitate pathological pain and opioid-insensitive status in the majority of studies (Uchida et al., 2010; Denk et al., 2013; Matsushita et al., 2013; Cherng et al., 2014). Accordingly, several kinds of HDAC inhibitors, including TSA, SAHA, sodium butyrate, MS-275, and LG325 exert analgesic effects in multiple pain models (Agudelo et al., 2011; Denk et al., 2013; Kukkar et al., 2014; Hou et al., 2017; Sanna et al., 2017; Danaher et al., 2018; Liao et al., 2018; Sanna and Galeotti, 2018; Zhao and Wu, 2018).

Due to lack of specific HDAC antagonists and the essential role of HDACs in embryo survival and development (Hou et al., 2017; Lin et al., 2017), only a few types of HDACs have been confirmed to contribute to neuropathic pain. Spinal nerve ligation provoked HDAC4 phosphorylation and resultant HDAC4 retention in the cytoplasm of dorsal horn neurons, which alleviated epigenetic suppression of the hmgb1 gene, and induced pain behaviors (Lin et al., 2015, 2016). In a cisplatin-induced peripheral neuropathy model, the HDAC6 inhibitor ACY-1083 prevented development of mechanical allodynia and completely attenuated established mechanical allodynia, spontaneous pain, and numbness. This effect was attributed to recovery of mitochondrial function in the dorsal root ganglia and nerve, and restoration of intraepidermal innervations by ACY-1083 (Krukowski et al., 2017). Recently, a highly selective HDAC1 inhibitor, LG325, was developed. LG325 completely reversed upregulation of HDAC1 within the spinal cord of SNI mice and ameliorated mechanical allodynia behavior (Sanna et al., 2017). HDAC1 and HDAC2 share approximately 80% amino acid homology and often form co-repressor complexes (Kramer, 2009). Previous studies reported the same trend of variation in HDAC1 and HDAC2 in bone cancer pain models (Hu et al., 2017; Hou et al., 2018). As such, HDAC2 may participate in neuropathic pain.

Unlike HDAC1 and class II HDACs, HDAC2 was primarily localized in the nucleus (Demyanenko et al., 2017; Zhu et al., 2017), suggesting a major role as a transcriptional regulator. In the brain, HDAC2 acts as a negative regulator of memory formation. Over-expression of HDAC2 impairs synaptic plasticity and memory, which may be due to modulation of neuron-specific genes by HDAC2 (Akhtar et al., 2009; Guan et al., 2009). Based on these results, focus on the role of HDAC2 in neurodegenerative diseases has increased (Yamakawa et al., 2017; Datta et al., 2018). In the spinal cord, HDAC2 was first identified as an essential regulator of oligodendrocyte development by forming a co-repressor with HDAC1 of beta-catenin-TCF interaction (Ye et al., 2009). Then, increased HDAC2 S-nitrosylation was reported in a CFA inflammatory pain model, and hence it was speculated to be involved in pain processing (Maiaru et al., 2016). Another study found that chronic fluoxetine treatment induced a sex-dependent analgesic effect, and this phenomenon may be mediated by HDAC2 repression and incremental mGlu2 receptor expression in the spinal cord dorsal horn of female mice (Zammataro et al., 2017). Recently, participation of HDAC2 in bone cancer pain and chronic pancreatitis pain was proposed, as a favorable pain-relieving effect was observed after HDAC2 knockdown or inhibition (Hu et al., 2017; Hou et al., 2018; Liao et al., 2018). In the present study, we found that HDAC2 mRNA and protein levels were both elevated after sciatic nerve constriction. Reversing this upregulation, whether by a chemical inhibitor or lentivirus introducing HDAC2-specific small-hairpin RNA, robustly alleviated mechanical and thermal hyperalgesia in CCI rats. It is noticeable that TSA suppressed the protein but not mRNA expression of HDAC2, possibly by promoting ubiquitination-mediated HDAC2 degradation, the similar phenomenon has been reported previously by Kramer et al. (2003) and Du et al. (2015). Besides that, our findings disagree with those from a study by Geranton et al., as they found that total expression of HDAC2 was unchanged in the lumbar spinal cord at the seventh day after spared nerve injury when tested by immunofluorescent staining. However, they also detected significantly increased HDAC2 signal in astrocytes of SNL rats (Maiaru et al., 2016). This discrepancy may due to the differences in animal models used, testing time points, and methods used for HDAC2 quantification.

Dysfunction of the inhibitory circuitry in the spinal cord underlies the central sensitization mechanism in chronic pathological pain. γ-aminobutyric acid (GABA) is the prominent inhibitory neurotransmitter in the dorsal horn. GABA is synthesized from glutamate by the enzyme glutamic acid decarboxylase (GAD) in inhibitory interneurons. There are two distinct isoforms of GAD, GAD67, and GAD65, encoded by *gad1* and *gad2*, respectively (Pinal and Tobin, 1998; Soghomonian and Martin, 1998). GAD65 is believed to be the isoform responsible for neuropathic pain (Moore et al., 2002; Zhang et al., 2011). After peripheral nerve injury (a neuropathic pain model), reduced GAD65 protein throughout the ipsilateral dorsal horn was detected (Moore et al., 2002; Lorenzo et al., 2014). Introduction of rAAV2-GAD65 to the ipsilateral DRG directly or through the sciatic nerve led to significant recovery in GAD65 expression in both ipsilateral DRG and spinal cord, which resulted in elevation of GABA concentration in the spinal cord and attenuated pain symptoms (Lee et al., 2007; Kim et al., 2009). Further study found that under persistent inflammatory and neuropathic pain, GAD65 was epigenetically suppressed with reduced H3 acetylation and enhanced recruitment of HDAC1, HDAC2, and HDAC4 to the gad2 promoter (Zhang et al., 2011). Administration of TSA, SAHA, or MS-275 dramatically restored GAD65 expression and alleviated pathological pain (Zhang et al., 2011; Shen et al., 2014). This analgesic effect was abolished in *gad65* knock-out mice (Zhang et al., 2011). Consistent with these findings, our data further confirmed the modulating activity of HDAC2 on GAD65 expression in the spinal cord.

GABA released by inhibitory interneurons binds to post-synaptic GABA receptors and generates inhibitory synaptic currents. This function relies on a transmembrane chloride gradient, which was maintained by cation-chloride cotransporters (CCCs) (Blaesse et al., 2009). KCC2 is a well-studied CCC ubiquitously expressed in the nervous system that mediates chloride extrusion driven by Na+-K+-ATPase, thus sustaining lower intracellular chloride concentration (Rivera et al., 1999). After peripheral nerve injury, the expression of KCC2 was decreased in the ipsilateral spinal cord. Knockdown or inhibition of KCC2 in intact rats induced hypernociceptive behavior similar to neuropathic pain (Coull et al., 2003). In multiple pathological pain conditions, enhanced KCC2 expression or function results in marked pain relief (Zhang et al., 2008, 2017; Gagnon et al., 2013; Sanchez-Brualla et al., 2018). In a study of persistent inflammatory pain, histone hypoacetylation status on the KCC2 promoter was responsible for its reduction. Although no HDAC tested was upregulated, long-term enhanced binding of HDAC1, 2, 5, 6, and 7 to the KCC2 promoter was detected (Lin et al., 2017). Our previous research showed restoration of spinal cord KCC2 expression after HDAC2-shRNA application in a bone cancer pain rat model (Lin et al., 2017; Hou et al., 2018) Consistent with these results, our present study showed that enhanced expression of KCC2 in the spinal cord after HDAC2 knockdown may account for the analgesic effect resulting from HDAC2 suppression.

Our study showed for the first time that HDAC2 in involved in the pathological process of neuropathic pain induced by peripheral nerve injury. GAD65 and KCC2 are possible downstream targets of HDAC2 in the pain-modulating pathway. Currently, several kinds of HDACs inhibitors have been successfully used in clinical treatment or clinical trials with relatively tolerable side effects (Eckschlager et al., 2017). Understanding the detailed mechanisms of HDACs subtypes in pain processing has been valuable in achieving more specific therapies and minimizing unintended disturbances to homeostasis. To further understand the role of HDACs in pain, changes in HDAC2 recruitment to the promoter of GAD65 and KCC2 and the effect of HDAC2 knockdown on electrophysiological function of inhibitory interneurons in the spinal cord needs to be confirmed. Other possible downstream targets of HDAC2 in pain processing, such as MOR, BDNF, and mGlu2 receptor could also be evaluated (Kurita et al., 2012; Hsiao et al., 2017; Liao et al., 2018).

## Ethics Statement

This study was carried out in accordance with the recommendations of guideline provided by the International Association for the Study of Pain (IASP). The protocol was approved by the Xiangya Medical College of Central South University Animal Care and Use Committee.

## Author Contributions

YW and QG designed and conceived the experiments. BO, DC, XH, and TW performed the experiments. WZ and YW analyzed the data. JW, CH, and ZS contributed reagents, materials, and analytical tools. YW and BO wrote the manuscript.

## Conflict of Interest Statement

The authors declare that the research was conducted in the absence of any commercial or financial relationships that could be construed as a potential conflict of interest.

## Supplementary Material

FIGURE S1 Suppressing HDAC2 by TSA **(A)** or LV-shRNA-HDAC2 **(B)** did not affect the motor function of rats.

FIGURE S2 The number of HDAC2 positive cells was increased time-dependently in the ipsilateral dorsal horn of CCI rats. Kruskal–Wallis test with Dunn’s post-test was used, *n* = 4 per group, ^∗^*p* < 0.05, compared with the sham group (Kruskal–Wallis statistic = 17.90, *P* = 0.0031; multiple comparisons: sham vs. D7, 316.8 ± 9.928 vs. 440.8 ± 13.89, *p* = 0.0296; sham vs. D14, 316.8 ± 9.928 vs. 462.5 ± 24.94, *p* = 0.0145; *n* = 4 for each group).

FIGURE S3 The immunohistochemical image showed an increase of HDAC2 in the ipsilateral dorsal horn (marked by arrow) of CCI rats at the 10th day after surgery.
