# Supplementary figures and images for: Normalizing HDAC2 Levels in the Spinal Cord Alleviates Thermal and Mechanical Hyperalgesia After Peripheral Nerve Injury and Promotes GAD65 and KCC2 Expression
Source: Front Neurosci. 2019 Apr 10;13:346. doi: 10.3389/fnins.2019.00346 (PMC6468568; doi:10.3389/fnins.2019.00346)

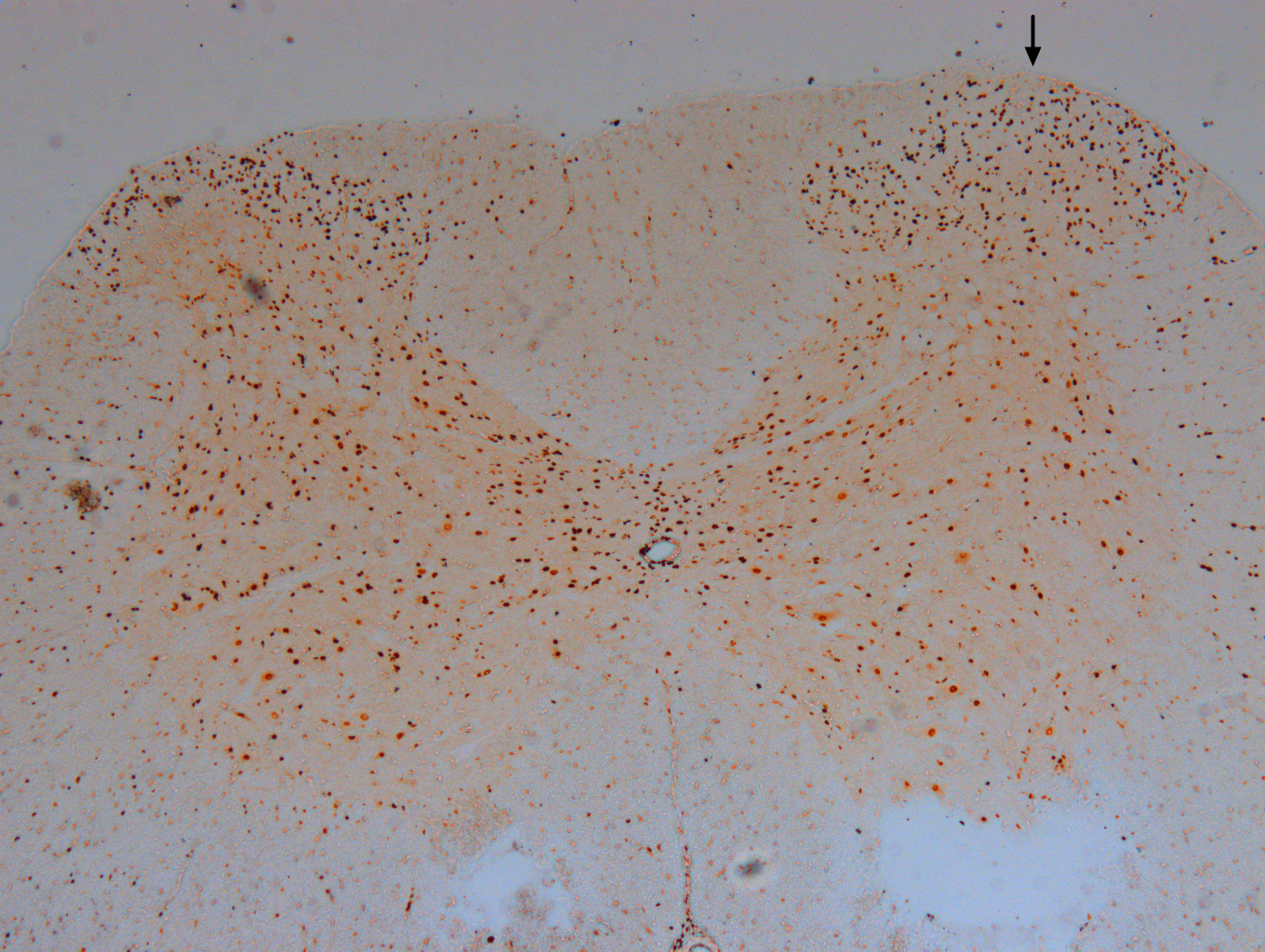

Supplement: FIGURE S3 — The immunohistochemical image showed an increase of HDAC2 in the ipsilateral dorsal horn (marked by arrow) of CCI rats at the 10th day after surgery. [file Image_3.tif]
